# Identification and characterization of protective CD8^+^ T‐epitopes in a malaria vaccine candidate SLTRiP

**DOI:** 10.1002/iid3.283

**Published:** 2020-01-22

**Authors:** Afshana Quadiri, Inderjeet Kalia, Mohammad Kashif, Agam P. Singh

**Affiliations:** ^1^ Infectious Diseases Laboratory National Institute of Immunology New Delhi India

**Keywords:** liver‐stage malaria, T‐cell epitopes, vaccine

## Abstract

**Introduction:**

Efforts are required at developing an effective vaccine that can inhibit malaria prevalence and transmission. Identifying the critical immunogenic antigens and understanding their interactions with host proteins forms a major focus of subunit vaccine development. Previously, our laboratory showed that SLTRiP conferred protection to the liver stage of *Plasmodium* growth in rodents. In the follow‐up of earlier research, we demonstrate that SLTRiP‐mediated protection is majorly concentrated in specific regions of protein.

**Method:**

To identify particular protective regions of protein, we synthesized multiple nonoverlapping fragments from SLTRiP protein. From this, we designed a panel of 8‐20mer synthetic peptides, which were predicted using T‐epitope‐based prediction algorithm. We utilized the IFN‐γ enzyme‐linked immunosorbent spot assay to identify immunodominant peptides. The latter were used to immunize mice, and these mice were challenged to assess protection.

**Results:**

The protective polypeptide fragment SLTRiP C3 and SLTRiP C4 were identified, by expressing and testing multiple fragments of PbSLTRiP protein. The immune responses generated by these fragments were compared to identify the immunodominant fragment. The T‐epitopes were predicted from SLTRiP protein using computer‐based algorithms. The in vitro immune responses generated by these peptides were compared with each other to identify the immunodominant T‐epitope. Immunization using these peptides showed significant reduction in parasite numbers during liver stage.

**Conclusion:**

Our findings show that the protective efficacy shown by SLTRiP is localized in particular protein fragments. The peptides designed from such regions showed protective efficacy equivalent to whole protein. The sequence conservation analysis with human *Plasmodium* species also showed that these peptides were conserved. In conclusion, these peptides or their equivalent from other *Plasmodium* species could impart protection against malaria in their respective hosts too. Our studies provide a basis for the inclusion of these peptides in clinical vaccine constructs against malaria.

## Graphical abstract

SLTRiP protective polypeptide fragment was identified by expressing and testing multiple fragments of PbSLTRiP protein and protective T‐epitopes were identified from a curated list of peptides. ELISpot assay was performed to identify the immunodominant T‐epitope. Sporozoite challenge assay done with immunized mice, was used to pinpoint protective CD8 + T‐epitopes.

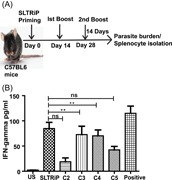

**Abbreviation**
*P. berghei, Plasmodium berghei*, PBS: phosphate‐buffered saline

## 1 INTRODUCTION

Malaria is a mosquito‐borne infectious disease affecting annually an estimated 212 million people worldwide, the causative agent being an Apicomplexan parasite of genus *Plasmodium*.^1^ The disease manifests in a broad range of clinical symptoms varying from moderate symptoms like fever and diarrhea to life‐threatening symptoms, which include severe anemia, respiratory distress, renal impairment, coma, and even death. Despite high mortality, no licensed vaccine that can provide 100% (sterile) protection against *Plasmodium* infection exists. The complicated genetic structure and high antigen diversity of *Plasmodium* make malaria vaccine generation a daunting task. The situation has become perilous with the increasing resistance of *Plasmodium* against common antimalarial drugs.^2^, ^3^ In fact, resistance against artemisinin has also been reported from various parts of Asia and Africa.^4^, ^5^ In addition, most known therapeutic drugs against *Plasmodium* restrict or kill parasites during its blood stage. The need for an effective vaccine against malaria that targets both blood as well as liver stage has become indispensable for the control and eradication of malaria.^6^ This is necessary as some *Plasmodium* species persist as dormant hypnozoites in the liver, which are activated anytime from days to years after primary infection, causing relapse of blood‐stage parasite.

The vaccines designed against microbes belong to one of the three categories—killed parasite, attenuated parasite, and subunit vaccines. Live radiation‐attenuated *Plasmodium berghei* sporozoites (RAS) were the first vaccines against malaria that gave full sterile protection against the challenge of live sporozoites and is considered the “gold standard” for development of malaria pre‐erythrocytic stage subunit vaccines.^7^, ^8^, ^9^, ^10^, ^11^ Immunization using chemically and or genetically attenuated malaria parasites have been shown to provide immunity against multiple strains of *Plasmodium* parasite.^12^, ^13^, ^14^ However, the approach faces the challenges of manufacture cost, storage, and distribution of parasite, thus limiting the use of this approach in endemic areas. Conversely, a subunit vaccine includes one or multiple protein antigen that may or may not be coupled to immunogenic and protective epitopes.

A very few subunit vaccines against different infectious diseases have been licensed and are being used. These include tetanus, diphtheria and pertussis (TDP) toxins, hepatitis B surface antigen, and vaccine against human papilloma virus.^15^ *Plasmodium* proteins have been assessed in murine models, for the development of therapeutic vaccines against vector‐ or host‐specific malarial stages. The synthesis of a peptide‐based vaccine called SPf66, with apparent efficacy against monkeys generated enormous interest for field trials in Africa to demonstrate protection.^16^ The studies with SPf66 also led to the development of field technologies to evaluate different vaccine candidates.

Malaria sporozoites express exoerythrocytic stage‐specific virulent proteins important for productive hepatocyte invasion. These include CSP, EXP1, TRAP, SPECT1, SPECT2, CelTOS, UIS4, and PPLP1 and many other proteins.^17^ These proteins have been studied for their protective efficacy, some of which, like circumsporozoite protein (CSP) and TRAP, are already in the advanced stages of vaccine development.^18^ The major sporozoite coat protein, CSP, is well characterized and widely used as a model antigen. The central repeat (R) region and the T‐cell epitopes (T) of *Plasmodium falciparum* CSP combined with hepatitis B surface antigen given along with the AS01 adjuvant system (RTS,S/AS01), provides partial protective immunity against malaria infection primarily through high levels of antibodies.^19^ The protection is limited to a maximum percentage of 40 to 50 and the antigen needs to be improved for its efficacy, by combining it with new antigens and adjuvants.^20^ The identification of *Plasmodium* surface protein‐circumsporozoite protein led to an optimistic prediction of a possible subunit vaccine against malaria. However, validation of the abilities of vaccine antigen candidates for boosting immune responses and providing 100% sterile protection in humans is still in process. Multiple tests are done to increase the efficacy of partially effective antigens through assessments of new adjuvants, delivery platforms, and/or identifying new candidate antigens. Epitopes from MSP, LSA‐1, and CSP have been tested alone and as part of multiepitope antigen.^21^ Therefore, epitope‐enhanced immunogens, expressing multiple copies of linear B and T‐cell epitopes from candidate antigens could be an important strategy to increase the protective efficacy of these vaccines.

In a previous work, we reported the protective efficacy of a novel antigen SLTRiP.^22^ SLTRiP immunization affected the growth of parasites within hepatocytes by delaying the prepatent period by 3 to 4 days. Immunized mice displayed protection subsequent to sporozoite challenge and exhibited 10 000‐fold less parasites 18S ribosomal RNA (rRNA) copy numbers in liver thus emphasizing the vaccine potential of SLTRiP.^22^ These data support the potential of SLTRiP as a target antigen for malaria vaccine development. In addition, this protection was majorly attributed to cell‐mediated immune system. In this paper, we aim to identify immunodominant and subdominant T‐cell epitopes, interferon γ (IFN‐γ) secretion by T cells against those epitopes. In addition, we attempted to identify epitopes involved in protection. Studies have shown that the T‐cell epitopes bind to peptide‐binding groove on MHC molecules, which has hydrophobic regions. To fit this groove, T‐cell epitopes need hydrophobic amino acids while hydrophilic regions are needed for interaction with T‐cell receptor. Bioinformatics approach to classify epitopes using Parker hydrophilicity prediction was employed to identify hydrophobic regions, which are likely to contain high‐scoring T‐cell epitopes.^23^ The study reports a protein antigen and its protective regions that can facilitate the development of a second‐generation vaccine against malaria.

## 2 MATERIALS AND METHODS

### 2.1 Ethics statement, experimental animals, and parasites

Six‐ to eight‐week‐old male/female C57BL/6 mice (H2^b^) were used in all animal experiments. The animal work was conducted in accordance with National Institute of Immunology's (NII) Institutional Animal Ethics Committee (IAEC) rules. The IAEC approval number for the project is NII‐312/13. Animals were injected with ketamine/xylazine intraperitoneally for short‐term anesthesia. At the end of each experiment, the anesthetized mice were killed humanely by cervical dislocation.

### 2.2 Parasite cycle

Six‐ to eight‐week‐old male/female C57BL/6 mice were used for growing parasites. *P. berghei* ANKA parasites were cycled between mice and *Anopheles stephensi* mosquitoes. Mosquitoes (3‐5 days old, female) were starved overnight and fed on infected mice. These infected mosquitoes were kept at 19°C, 70% to 80% relative humidity, 12 hours light cycle and fed on cotton pads soaked in 20% sucrose solution for 18 days post infected blood meal. After 18 days, sporozoites were obtained from dissection of salivary glands from infected mosquitoes. For this, the infected mosquitoes were first washed with 50% ethanol, followed by PBS, and dissected in RPMI 1640 media containing 10% fetal bovine serum. To obtain sporozoites, salivary glands were ground gently and centrifuged at 800 rpm for 4 minutes to remove mosquito tissue. The number of sporozoites present in per unit volume (mL) was determined by counting in a hemocytometer.

### 2.3 Bioinformatics analysis

Parker hydrophilicity prediction was used to distinguish between the hydrophilic and hydrophobic regions of the protein. The region above the threshold value are generally hydrophilic (shown in yellow) while the regions below the threshold are hydrophobic (shown in green). The epitopes of SLTRiP were predicted using Immune Epitope Database (IEDB) analysis resource, http://tools.immuneepitope.org/mhci. This tool takes an amino acid sequence, or a set of sequences to determine possible MHC class I binding peptides. It establishes the probability of a particular amino acid sequence to form a T‐cell epitope by assigning a score or percentile rank. The lower the assigned score to a particular amino acid sequence, the greater is the probability of that region to form T‐cell epitopes. The prediction method allows choosing from a number of MHC class I binding prediction methods. Based on the availability of predictors and formerly observed predictive performance, this selection uses the best possible method for a given MHC molecule. Currently for peptide:MHC‐I binding prediction, for a given MHC molecule, IEDB Recommended uses the Consensus method consisting of ANN, SMM, NetMHCpan, and CombLib methods. We employed IEDB recommended 2.19 for our epitope prediction. The epitope predictions were limited to peptides of H2^b^ allele and specific to MHC class I. Variable‐length peptides were chosen based on position in protein and percentile rank.

### 2.4 Primer designing

The amino acid sequence of SLTRiP gene of *P. berghei* was scanned for hydrophobic regions. Primers were designed from hydrophilic region such that four fragments incorporating the whole gene were generated with no overlapping regions. Primers with *Bam*HI and *Xho*I sites were used to facilitate cloning of fragments in pGEX6P1 vector and express fragments of open reading frame of SLTRiP fused in‐frame to the 3′ end of the glutathione *S*‐transferase (GST) protein.

### 2.5 Expression and purification of SLTRiP fragments

The individual polypeptide fragments (C2, C3, C4, C5) were induced by addition of isopropyl 1‐thio‐d‐galactopyranoside (IPTG) to a final concentration of 1 mM when the bacterial culture reached 0.5‐0.7 OD_600_, followed by incubation of 12 hours at 37°C for C2; 18 hours at 18°C for C3 and C4; and 18 hours at 25°C for C5. The cells were harvested at 8000 rpm for 10 minutes at 4°C and suspended in buffer A (100 mM Tris, 250 mM NaCl, 10% glycerol, 0.5 mM EDTA, 0.05% Triton X‐100, pH 8.0) with 0.02 mg/mL lysozyme and protease inhibitor mixture to make complete lysis buffer. The suspension was sonicated at 4°C (ice‐cold) for 10 minutes. The sample was cleared by centrifugation at 12 000 rpm for 20 minutes at 4°C. The supernatant obtained was loaded onto a prepacked 5‐mL GST‐FF column and washed with 10 column volumes of buffer A. The protein was eluted with buffer A containing 15% v/v of buffer B (50 mM reduced glutathione) using AKTA explorer chromatography system. The purity was observed to be more than 95% in case of C3, C4, and C5. An additional step of gel filtration chromatography was employed for purification of C2. Yields were typically in the range of 3 to 4 mg of purified protein/L of bacterial culture for C2 and C5 but 0.5‐1 mg/L for C3 and C4.

### 2.6 Immunization with purified SLTRiP polypeptide fragments and SLTRiP peptides

C57BL/6 mice, aged 6 to 8 weeks were immunized; priming was done with 50 µg of polypeptide in complete Freund's adjuvant (Sigma, India) per mouse. In the three subsequent boosters, the amount of polypeptide used was 25 µg per mouse mixed with incomplete Freund's adjuvant (Sigma). Boosts were given on days 15, 21 and 28 post‐priming. The control group was immunized in an identical manner with GST protein.

### 2.7 Peptide synthesis

The studies were initially carried out with a panel of long protein fragments spanning the complete sequence of the SLTRiP protein. Later 15 peptides consisting of 9 to 16 amino acids, spanning the protective protein fragments were used to define minimal T‐cell epitopes. The peptides were synthesized commercially by Bio Basic (Canada) at more than 80% purity.

### 2.8 Enzyme‐linked immunosorbent assay

Culture supernatants from in vitro stimulated splenocytes were collected after 60 hours of incubation. Secreted cytokines were measured by enzyme‐linked immunosorbent assay (ELISA) using an eBiosciences kit, following manufacturer's instructions. The purified anticytokine antibody was added to the wells of enhanced protein binding ELISA plate, sealed, and incubated at 4°C overnight. The next day, the antibody solution was removed and the plate was blocked using blocking buffer for 1 to 2 hours at room temperature (RT) to prevent nonspecific binding. Plate was washed three times with PBST (1× phosphate‐buffered saline with Tween detergent). Biotinylated anticytokine detection antibody was added, sealed, and the plate was incubated at RT for 1 hour. It was washed again three times with 1× PBST. Secondary antibody conjugated with HRP was added to the wells, sealed, and incubated again at RT for 30 minutes. The plate was washed five times with PBST and developed using TMB (3,3′,5,5′‐tetramethylbenzidine) substrate until color starts to appear. Optical density was measured at 450 nm in a microplate reader (Tecan M200, UK).

### 2.9 Ex vivo IFN‐γ enzyme‐linked immunosorbent spot

Ex vivo enzyme‐linked immunosorbent spot (ELISpot) assay was done for peptide‐stimulated splenocytes following manufacturer's (BD Biosciences) protocol. Capture antibody diluted in coating buffer was added to each well of an ELISpot plate and stored at 4°C. Next day, antibody was discarded; plate washed and blocked with a blocking solution for 1 to 2 hours at RT. Splenocyte suspension was prepared and added at different dilutions (10^5^‐10^6^cells/mL) to wells of ELISpot plate. The cells were activated using proper mitogen and antigen. ELISpot plate was incubated at 37°C, in a 5% CO_2_ and humidified incubator for 24 hours. The cell suspension was aspirated and plate was washed three times with wash buffer. Detection antibody was prepared and added to ELISpot plate. The plate was incubated at RT for 2 hours, followed by washing three times again with wash buffer. Secondary Ab‐HRP enzyme conjugate was added to the plate and incubated for 1 hour at RT. The plate was washed five times with wash buffer and finally, substrate solution was added to each well of ELISpot plate. Spot development was monitored for 5 to 60 minutes and reaction was stopped, by washing wells with deionized water to prevent overdevelopment of spots, else it may give high background. The plate was air‐dried at RT for 2 hours or overnight until it was completely dry. The plate was stored in a sealed plastic bag in the dark, until analysis. Spots were enumerated using an ELISpot plate reader (AID iSpot ELHR04, Germany).

## 3 RESULTS

### 3.1 Generation of polypeptide fragments

**Figure 1:**
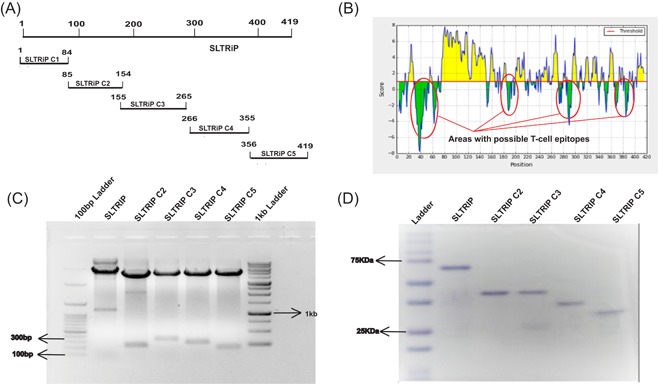
Construction of gene fragment clones and expression in *Escherichia coli*. A, Gene fragment clones were constructed from the SLTRiP gene, which was codon‐optimized for expression in *E. coli*, and the fragments were labeled as SLTRiP C2, SLTRiP C3, SLTRiP C4, and SLTRiP C5. B, Parker hydrophilicity prediction was done to identify hydrophobic regions, likely to contain T‐cell epitopes. Primers were designed from the gene corresponding to the hydrophilic regions of the protein. C, The individual gene fragments were amplified using SLTRiP codon‐optimized gene as template and were cloned in pGEX6P1 vector. D, The individual gene fragments were expressed and purified as glutathione *S*‐transferase (GST)‐tagged protein or protein fragments using the GST‐binding column

We aimed to identify immunodominant and subdominant T‐cell epitopes involved in protection against sporozoite challenge. For this, gene fragment clones were designed as an approach to predict the minimal protective region in SLTRiP (Figure 1A). Parker hydrophilicity prediction method was employed to distinguish hydrophobic regions from hydrophilic regions. We observed the regions designated as SLTRiP C1, SLTRiP C3, SLTRiP C4, and SLTRiP C5 had hydrophobic regions but SLTRiP C2 was majorly hydrophilic in nature (Figure 1B). The protein SLTRiP, mentioned in the work or used for immunization experiments starts from N‐terminus, amino acids 85 to 413. The exon 1, referred to as fragment SLTRiP C1 was added in later annotations to the protein and the peptides from this fragment have been used as negative control in peptide stimulation studies. The protein hydrophilic regions were used to design primers that corresponded to individual polypeptide fragment. This was done to ensure that none of the possible T‐cell epitopes are fragmented. The individual gene fragments were amplified using, *Escherichia coli* codon‐optimized SLTRiP gene as template and PCR primers (Table 1). The gene fragments were cloned in the bacterial expression vector pGEX6p1, which contains a GST tag at its N‐terminus (Figure 1C). The clones were confirmed and the individual gene fragments were expressed as GST‐tagged fusion protein or protein fragments. The fusion protein fragments were purified using the GST‐binding column. The purity of all fragments was observed to be above 95% from sodium dodecyl sulfate‐polyacrylamide gel electrophoresis image (Figure 1D). SLTRiP C2 fragment being majorly hydrophilic, gave yield in the range of 2 to 3 mg of purified protein per liter of the bacterial culture. The other fragments give yield in the range of 1 mg/L of bacterial culture, one of the reasons for low yield being the continuous stretches of hydrophobic amino acids as observed in Parker hydrophilicity prediction. The purified fragments qualified for subsequent analysis of their abilities to stimulate splenocytes.

**Table 1:** Primers for SLTRiP fragment cloning

Primer designation	Primer sequence, 5′‐3′
SLTRiP‐C2FP	CGAT‐**GGATCC**‐GAATACAGCGACGATGAGTA‐3
SLTRiP‐C2RP	TCAT‐**CTCGAG**‐AGAGATCACGCTGGATTTGC‐3
SLTRiP‐C3FP	CGAT‐**GGATCC**‐TTCATCAAAAAAAAACCGACGA‐3
SLTRiP‐C3RP	TCAT‐**CTCGAG**‐CAGGTTGTTCCAACGGTTG‐3
SLTRiP‐C4FP	CGAT‐**GGATCC**‐CAGACCGAAAACGAAAACAAC‐3
SLTRiP‐C4RP	TCAT‐**CTCGAG**‐CTCAATGTTGTAGATGTAGTG‐3
SLTRiP‐C5FP	CGAT‐**GGATCC**‐ATCCTGTGCAACAAAGAGAA‐3
SLTRiP‐C5RP	TCAT‐**CTCGAG**‐GATGTTAGAGCGTTCGCTAG‐3

*Note*: Restriction sites are shown in bold letters.

### 3.2 Identification of immunodominant SLTRiP fragment

**Figure 2:**
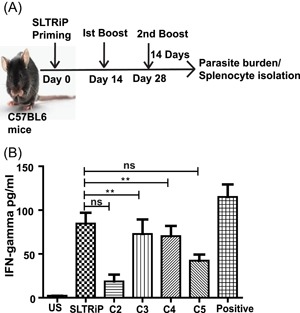
Identification of the immunodominant polypeptide. C57BL/6 mice aged 6 to 8 weeks were immunized with 25 μg of SLTRiP protein. A, The boosts were given after 2 weeks and the spleen was isolated, 2 weeks after the last boost. The splenocytes were stimulated in vitro with individual protein fragments for 2 days, in the presence of interleukin‐2 (IL‐2). The cell culture supernatants were collected and levels of interferon‐γ (IFN‐γ) were determined by enzyme‐linked immunosorbent assay method. All data are means and standard errors based on six mice per group. B, **P < .01; using one‐way analysis of variance Bonferroni's multiple comparison test. Positive, positive control stimulated with cell stimulation cocktail (Invitrogen); SLTRiP, mice stimulated with SLTRiP‐full; C2, C3, C4, and C5, sets of mice stimulated separately with polypeptides (SLTRiP fragments) in vitro; US, unstimulated

Studies have shown the role of cytokines in modulating immune responses. To demonstrate the most immunodominant fragment in terms of IFN‐γ secretion, an in vitro IFN‐γ ELISA was conducted. For this, the mice were immunized with SLTRiP protein (Figure 2A). The splenocytes from SLTRiP‐immunized mice were cultured and stimulated with individual fragments/polypeptides in vitro. The supernatant of culture was collected and quantitated for IFN‐γ concentration. The cultures stimulated with SLTRiP C2 and SLTRiP C5 showed IFN‐γ secretion on stimulation, which was more than the control. However, significantly increased secretion of IFN‐γ was observed in cultures stimulated with SLTRiP C3 and SLTRiP C4, which was comparable with that of SLTRiP protein stimulation (Figure 2B), establishing these two as immunodominant polypeptides/fragments.

### 3.3 SLTRiP fragments immunization and sporozoite challenge assay

**Figure 3:**
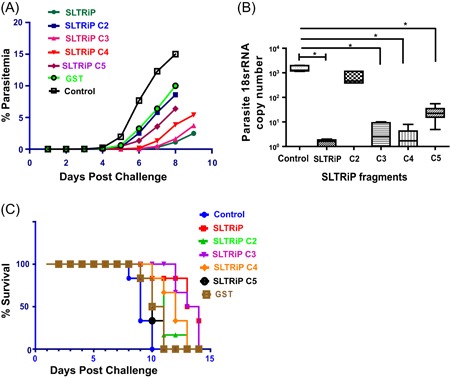
SLTRiP fragments immunization and sporozoite challenge assay: (A) the Giemsa‐stained slides showing the prepatent period of the parasite in mice immunized with SLTRiP and its fragments. The exoerythrocytic parasite burden (parasite 18S ribosomal RNA [rRNA] copy number) was quantified from the liver of mice immunized with SLTRiP or its fragments and challenged with wild‐type sporozoites. B, The 18S rRNA copy numbers were not normalized with mice glyceraldehyde‐3‐phosphate dehydrogenase (GAPDH) (control), as GAPDH values were equal in all the samples. C, The survival of immunized mice was calculated and plotted. The data are presented as means and standard error of the means of six mice per group. *P < .05; using one‐way analysis of variance Kruskal‐Wallis test. Control, immunized with adjuvant only; GST, immunized with GST protein; SLTRiP, immunized with SLTRiP; C2, C3, C4 and C5, sets of mice immunized separately with SLTRiP fragments

In the next set of experiments, we compared the protective efficacy of SLTRiP fragments by mice immunization experiment. Groups of 12, females 6‐week‐old C57BL6 mice were immunized with SLTRiP fragments subcutaneously as shown in Figure 2A. Two weeks after the last boost, the mice were challenged with 5000 *P. berghei* ANKA sporozoites given intravenously. One set of mice was analyzed for parasite load in mice liver while the other set of mice (six mice) was monitored for emergence and growth of blood‐stage parasite. The parasitemia levels were observed by microscopic examination of Giemsa‐stained thin blood smears prepared from day 3 post challenge and followed until the mice died. The parasitemia count for SLTRiP C3 and C4 showed a delay in prepatent period of 4 and 3 days while SLTRiP C2 and C5 showed a delay of 0 and 2 days, respectively. The prepatent delay shown by SLTRiP C3 and SLTRiP C4 was comparable with full‐length SLTRiP protein immunized mice (Figure 3A). Furthermore, a 3 log reduction in parasite 18S rRNA copy numbers was observed in mice immunized with SLTRiP C3 and SLTRiP C4; which is close to the reduction observed with SLTRiP protein. SLTRiP C5 showed nearly 2 log reduction, while SLTRiP C2 showed less than 0.5 log reduction in mice liver burden (Figure 3B). The survival assay showed an increased survival of 4 days in mice immunized with SLTRiP C3 and SLTRiP C4 while an increased survival of only 1 day was observed in SLTRiP C2‐ and C5‐immunized mice compared with control (Figure 3C). Overall, the sporozoite challenge assay showed a decrease in parasite load, delay in prepatent period, and increased survival in mice immunized with SLTRiP C3 and SLTRiP C4, which was comparable with SLTRiP protein. These results indicate that the protection contributed by SLTRiP is located mostly in these fragments (C3 and C4) of SLTRiP.

### 3.4 Immunogenicity and protective efficacy of putative T‐cell epitopes

**Figure 4:**
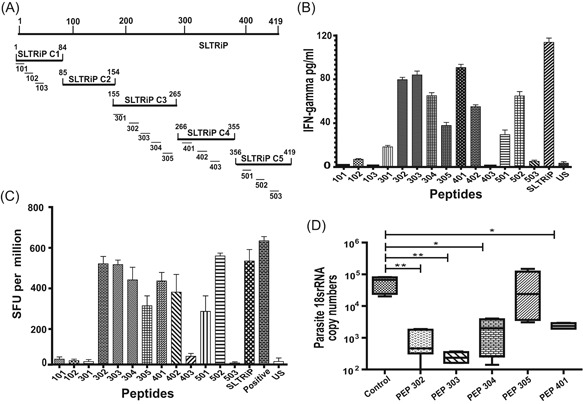
SLTRiP T‐cell epitope peptides characterization: splenocytes were collected from the immunized mice, cultured in presence of IL‐2, and stimulated with peptides at concentration 10 ng/well for 3 days. A, The comparative location of peptides in protein is shown. B, Epitope‐induced interferon‐γ (IFN‐γ) secretion was monitored by IFN‐γ enzyme‐linked immunosorbent assay. C, Epitope‐induced IFN‐γ spot formation was monitored by enzyme‐linked immunosorbent spot assay by calculating the number of spots formed in each stimulated well. The decrease in pre‐erythrocytic parasite burden (parasite 18S rRNA copy number) in the liver of mice immunized with peptides and challenged with wild‐type sporozoites.was quantified. D, The 18S rRNA copy numbers were not normalized with mice glyceraldehyde‐3‐phosphate dehydrogenase (GAPDH) control as the values for all GAPDH copy numbers were equal in all the samples. The data represented are means and standard error of the means based on six mice per group. *P < .05; **P < .01; by one‐way analysis of variance Kruskal‐Wallis test. Control, mice immunized with adjuvant; PEP, peptide

Bioinformatics approaches to identify T‐cell epitopes, have been used in many infectious diseases for their inclusion in vaccines with success. Peptides joined as a string of beads were synthesized as a recombinant protein to immunize against *P. falciparum* epitopes. In this study, bioinformatics approach was used to screen for potential T‐cell epitopes in SLTRiP and to identify T‐ epitopes with the potential to provide protection against *P. berghei* sporozoite challenge in mice. In this regard, 8‐20mer consecutive peptides that encompass the entire protective gene fragments were synthesized using T cell‐based algorithm. Table 2 shows the list of all peptides chosen from SLTRiP for use in in vitro stimulation and protection assay. IEDB tool was used for T‐epitope prediction. Epitopes from SLTRiP fragments namely C1, C3, C4, and C5 were predicted using IEDB analysis and chemically synthesized. Their comparative location in protein is shown in Figure 4A.

**Table 2:** The table shows the list of peptides selected from protective fragments for in vitro stimulation and in vivo protection assay

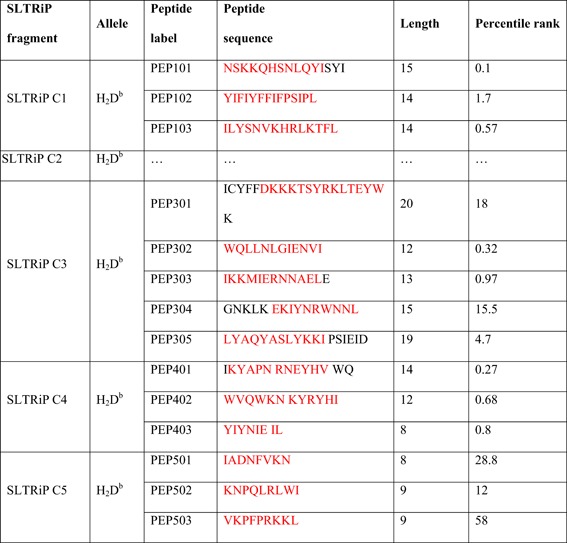

*Note*: Peptide length and percentile rank is given in the rightmost columns. IEDB recommended 2.19 was used for our epitope prediction. The percentile rank given corresponds to the sequences in red. The lower the assigned score to a particular amino acid sequence, the greater is the probability of that region to form T‐cell epitopes.

Splenocytes were collected from SLTRiP‐immunized mice and incubated with peptide at concentration 10 ng/well in a 96‐well plate for 72 hours. Epitope‐induced IFN‐γ secretion was monitored by IFN‐γ ELISA. An increase in the release of IFN‐γ was observed in cells stimulated with peptides 302, 303, 304, and 401 (Figure 4B). A suboptimal increase was also observed in cells stimulated with peptides 305, 402, and 502. Similarly, splenocytes collected from SLTRiP‐immunized mice were incubated with peptides at concentration 10 ng/well for 24 hours in an ELISpot plate. Epitope‐induced IFN‐γ secretion was monitored by the formation of spots on membrane. An increase in number of IFN‐γ spots was observed in wells stimulated with peptides 302, 303, 304, 401, and 502 (Figure 4C), while comparatively moderate spots were also observed in wells stimulated with peptides 305 and 402. These results determined peptides 302, 303, 304, 401, and 502 as immunodominant peptides, and 305 and 402 as subdominant peptides. As fragment analysis had shown that most of SLTRiP‐related protection is concentrated in fragment SLTRiP C3 and SLTRiP C4; peptides 302, 303, 304, 305, and 401 were further used for immunization to analyze them for their protective efficacy.

**Figure 5:**
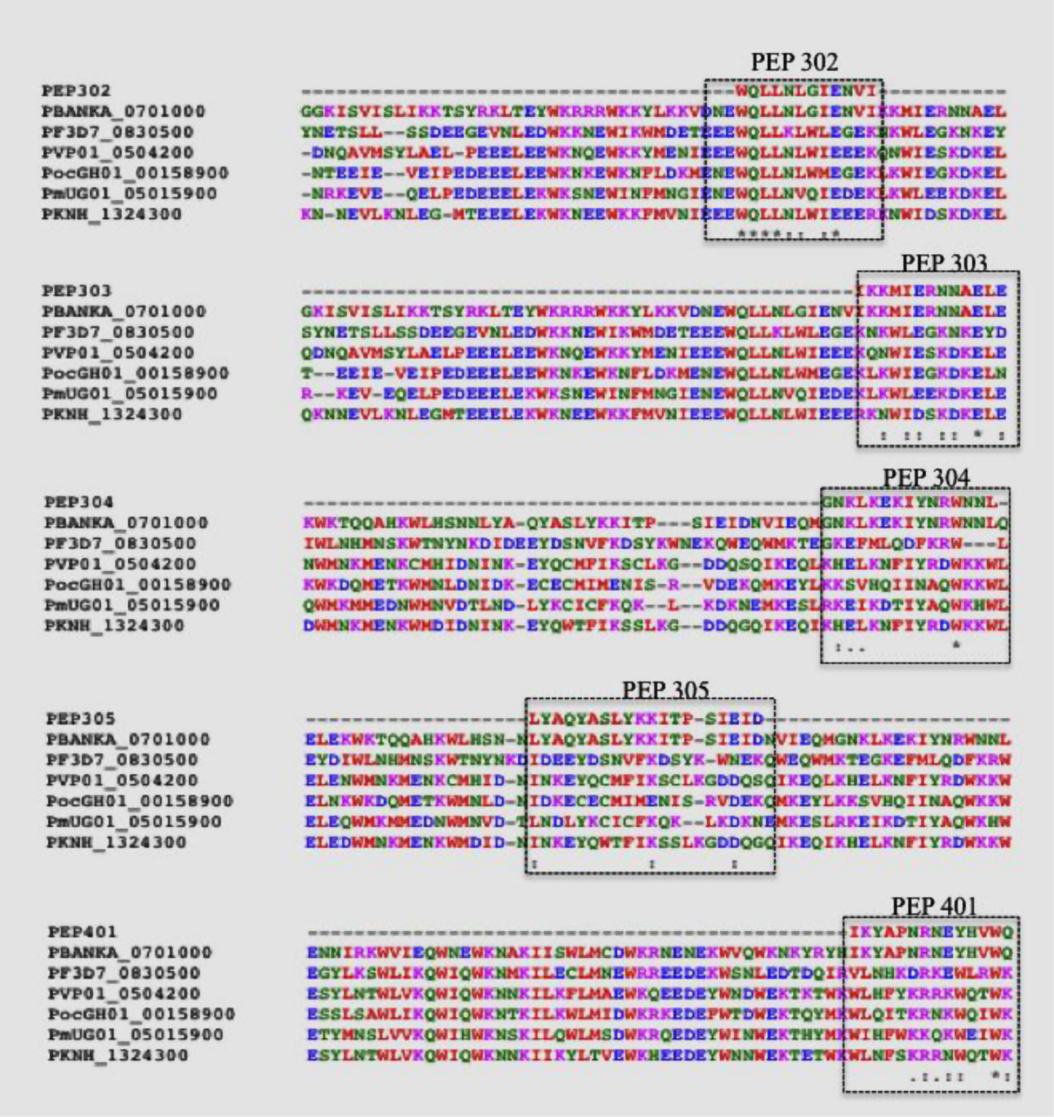
T‐epitope conservation among human parasites. Multiple sequence alignment of SLTRiP immunodominant peptides (302, 303, 304, 305, and 401) with SLTRiP orthologs in human *Plasmodium* species: *P. falciparum* (PF3D7_0830500), *P. vivax* (PVP01_0504200), *P. ovale* (PocGH01_00158900), *P. malariae* (PmUG01_05015900), *P. knowlesi* (PKNH_1324300). The conservation of amino acid tryptophan (W), along with other hydrophobic amino acids (V, I, L, F) and charged amino acids (K, R, E, D) is majorly observed in protective peptides 302 and 303. “*” fully conserved residue; “:” conservation between strongly similar residues; “.” conservation between weakly similar residues

Groups of C57BL/6 mice (5‐6 mice/group) aged 6 to 8 weeks were immunized with above mentioned immunodominant peptides. The immunization schedule was same as shown in Figure 2A and 50 or 25 μg of peptides were used for priming and boosts, respectively. A week after the final boost, mice were challenged with 10 000 *P. berghei* ANKA sporozoites given via intravenous route. The burden of parasite in liver was quantified by measuring parasite 18S rRNA using real‐time PCR analysis. PEP 302 showed 2 log reduction and PEP 303 showed 2.5 log reduction in parasite 18S rRNA copy numbers while PEP 304 and PEP 401 showed only 1.5 log reduction. PEP 305 showed no reduction in mice liver burden as compared with control immunized mice. (Figure 4D). The significant reduction in parasite burden in PEP 302‐ and PEP 303‐immunized mice confirmed these peptides as dominant protective epitopes (Figure 5).

### 3.5 T‐epitope conservation among the human parasites

To identify the conservation of T‐epitopes, the sequences of these immunodominant peptides were aligned with homologous proteins in human *Plasmodium* species using ClustalW tool. The aligned sequences showing identical amino acids with no gaps were considered conserved across species. PEP 302 showed nearly 90% conservation in amino acids across species followed by PEP 303 which also showed considerable amino acid conservation. Furthermore, the protective T‐epitopes (peptides 302, 303, 304, and 401) contained the positionally conserved tryptophans (W), which is one of the features of SLTRiP protein. Besides tryptophan, other hydrophobic amino acids (V, I, L, F) and charged amino acids (K, R, E, D) conservation is evident in protective T‐epitopes (Figure 5). Nonprotective T‐epitope (peptide 305) lacks the conservation of amino acids observed in protective T‐epitopes.

## 4 DISCUSSION

The current research on a malaria subunit vaccine is majorly built on recombinant sporozoite protein, RTS,S. A number of alternatives exist for inducing immune responses, which include attenuated or inactive parasites. Although considerable efforts are being made at overcoming the limitations associated with irradiated and genetically attenuated sporozoites, the approach of formulating parasite recombinant proteins in an adjuvant, ought to be explored. Furthermore, there exists a general notion that multiple antigens, either through whole‐parasite inclusion of many proteins or polypeptides are required for an effective malaria vaccine.^24^ However, protein fragments are unlikely to be effective unless they include critical epitopes recognized by protective immune cells. An approach for development of effective vaccine against malaria includes identification of protein regions that can generate protective immune responses.^25^ Epitope mapping has multiple advantages for vaccine development as they represent antigenic regions of the protein and consequently nonprotective parts can be removed.^26^, ^27^ Epitopes from different alleles can also be collected for the formation of a peptide library that will be recognized by majority of immune populations. In addition, using mouse model, the peptide‐specific protective efficacy can be characterized by mice immunization.

SLTRiP protein was of particular interest as it was able to demonstrate protective efficacy in mice model.^22^ The in silico analysis showed that protein has B‐ and T‐cell epitopes. An epitope is frequently used for an immunodominant peptide. Our previous results demonstrate that the high titer antibodies generated were nonprotective. Therefore, we proceeded to identify T‐cell epitopes in SLTRiP. For this, we synthesized multiple nonoverlapping fragments from SLTRiP protein to identify particular protein fragments that are protective. Identification of the T‐cell epitopes of the 413 amino acid long SLTRiP protein would have been a laborious process by conventional methods, as it involves synthesizing short overlapping oligopeptides of the full‐length protein. We, therefore, synthesized multiple fragments of our gene to identify protective regions. The in silico studies have shown that the epitopes for T‐cells are generally present in hydrophobic regions of protein while hydrophilic regions score best for B‐cell epitopes.^27^ Using this information, bioinformatics approach was employed to identify hydrophobic regions of the protein using Parker hydrophilicity prediction to identify hydrophobic regions in protein. By generating recombinant subfragments of the protein SLTRiP, we observed that the immunodominant T‐cell epitopes of the protein are located between amino acids 155 and 355, which forms the protective SLTRiP C3 and SLTRiP C4 fragments of protein SLTRiP. These two fragments demonstrated major protection in C57BL/6 mice, which are conventionally difficult to protect than other mice strains.^28^ Mice immunized with SLTRiP C5 showed partial protection while SLTRiP C2 showed no protection. SLTRiP C2 contains mostly hydrophilic regions of protein as seen by Parker hydrophilicity prediction. The nonprotective results of C2 correspond with our earlier hypothesis stating that most of protection relies on T‐cell epitopes.

To study the protective epitopes, first of all T‐epitopes from the protective fragment were predicted using computer algorithm‐based predictions. These programs predict the potential of a peptide to bind to a particular MHC class I molecule using MHC peptide binding. Number of peptides required to be synthesized could be significantly reduced by employing such methods. The program prediction is based on the affinity of the peptides and T‐cell receptor but it cannot predict the processing, proteolysis, expression, or availability of the peptide on the cell surface. Some of the peptides predicted do not induce a T‐cell proliferation response and the peptides need to be checked in vitro for their ability to induce immune responses. Previous research has shown that protective immune responses after immunization are dependent on T cells secreting IFN‐γ.^29^ Therefore, we identified and validated T‐cell epitopes of the SLTRiP protein immunodominant for IFN‐γ secretion; however, these may be only a subset of the total T‐cell repertoire that exists in vivo.

Recently, the whole‐parasite vaccination approach has also been revived, despite challenges in sporozoite production. A number of blood‐stage and transmission‐blocking candidates are also being tested with different adjuvant formulation and delivery routes for malaria vaccine development; however, many groups believe that evaluation and identification of subunit vaccine candidates, acting synergistically to induce protective responses, can add to the efforts targeting multiple stages of parasite's life cycle. The subunit vaccines undergoing clinical assessment currently are PfCSP and PfTRAP. Studies in mice have shown that immunization using viral vectors expressing ME‐TRAP (multiepitope TRAP) induces protective immune responses in the liver.^30^ Although SLTRiP peptides do not provide sterile protection preclinically, we demonstrate the significantly high level of protective efficacy of these peptides by immunization and challenge experiments. In addition, sequence conservation analysis with human *Plasmodium* species revealed that these peptides were conserved, in fact, some amino acid residues, particularly positionally constrained tryptophan showed 100% identity in most of these strains. Therefore, these peptides or its equivalent from other *Plasmodium* species could impart protection against malaria in other hosts too. While antigens like CSP provide greater levels of protection clinically, we demonstrate the value of including these peptides in a multicomponent subunit second‐generation vaccines for the development of a subunit vaccine with improved protective efficacy.

## CONFLICT OF INTERESTS

The authors declare that there are no conflict of interests.

## Data Availability

All data are shown within the manuscript and figures. The raw data and the analysis details that support the findings of this study are available from the corresponding author upon reasonable request.
